# Plant-associated bacteria and their role in the success or failure of metal phytoextraction projects: first observations of a field-related experiment

**DOI:** 10.1111/1751-7915.12038

**Published:** 2013-02-20

**Authors:** Nele Weyens, Bram Beckers, Kerim Schellingen, Reinhart Ceulemans, Sarah Croes, Jolien Janssen, Stefan Haenen, Nele Witters, Jaco Vangronsveld

**Affiliations:** 1 Hasselt University, Centre for Environmental SciencesAgoralaan Building D, 3590, Diepenbeek, Belgium; 2 University of Antwerp, Biology DepartmentCampus Drie Eiken, Universiteitsplein 1, 2610, Wilrijk, Belgium

## Abstract

Phytoextraction has been reported as an economically and ecologically sound alternative for the remediation of metal-contaminated soils. Willow is a metal phytoextractor of interest because it allows to combine a gradual contaminant removal with production of biomass that can be valorized in different ways. In this work two willow clones growing on a metal-contaminated site were selected: ‘Belgisch Rood’ (BR) with a moderate metal extraction capacity and ‘Tora’ (TO) with a twice as high metal accumulation. All cultivable bacteria associated with both willow clones were isolated and identified using 16SrDNA ARDRA analysis followed by 16SrDNA sequencing. Further all isolated bacteria were investigated for characteristics that might promote plant growth (production of siderophores, organic acids and indol acetic acid) and for their metal resistance. The genotypic and phenotypic characterization of the isolated bacteria showed that the TO endophytic bacterial population is more diverse and contains a higher percentage of metal-resistant plant growth promoting bacteria than the endophytic population associated with BR. We hypothesize that the difference in the metal accumulation capacity between BR and TO clones might be at least partly related to differences in characteristics of their associated bacterial population.

## Introduction

Industrial and agricultural activities, together with urbanization and military operations resulted in a pollution of the environment by metals and organic contaminants that is nowadays a problem of global concern. Only Europe counts for up to 3.6 million potentially contaminated sites (EU Commission staff working document, 2006). This extent of contamination causes a burden on agricultural revenues (Meers *et al*., 2010), regional policy (Flemish Government, 2007), public health (Nawrot *et al*., 2006) and the self-cleansing capacity of polluted ecosystems (Doty, 2008). Contaminated soils and groundwater can be remediated by a wide range of technologies. However, in case of metal contamination, only a few technologies can be applied because of the immutable and rather immobile character of metals (Vangronsveld and Cunningham, 1998). Beside of their high costs, conventional remediation strategies destroy soil structure and biological activity in soils, meaning that, after treatment, they cannot be used for agricultural purposes (Vangronsveld and Cunningham, 1998). For these reasons, the need for inventive, sustainable and at the same time effective remediation technologies for large-scale metal-contaminated sites has never been greater.

The last two decades, plant-based technologies (generally termed ‘phytoremediation’) have emerged as valuable and sustainable alternatives to conventional remediation techniques (Barac *et al*., 2009; Vangronsveld *et al*., 2009). In general, phytoremediation is considered to be a cost-effective, eco-friendly, sustainable, risk managing technology that even offers the possibility of economic (non-food) activity and in many cases provides economic valorization potential (e.g. biofuel) (Vassilev *et al*., 2004; Vangronsveld *et al*., 2009; Witters *et al*., 2009; 2012a,b). As metal phytoextraction projects often require quite long periods of time (van der Lelie *et al*., 2001; Koopmans *et al*., 2007; Maxted *et al*., 2007; Vangronsveld *et al*., 2009; Ruttens *et al*., 2011), they need to integrate an attractive economic alternative to local farmers. The production of bio-energy crops on metal-contaminated land would provide the farmers with an income and in the meanwhile allow a sustainable use and valorization of the contaminated land (Van Ginneken *et al*., 2007; Weyens *et al*., 2009b; Thewys *et al*., 2010a,b; Witters *et al*., 2012a,b). On top of that, the production of biofuel and industrial feedstocks on marginal land that is not suited for agriculture can be an approach to moderate the conflict between food and energy crops (Weyens *et al*., 2009b).

In the North East of Belgium and in the South of the Netherlands (Campine region), an enormous region (700 km^2^) got enriched with several metals as a result of past activities of different pyrometallurgical zinc smelters (Vangronsveld *et al*., 1995; Hogervorst *et al*., 2007). As the region mainly consists of slightly acidic (pH 4–6) sandy and sandy-loam soils, a high metal mobility and plant availability is observed. This entails an enhanced risk for uptake of these metals in crops resulting in food and fodder crops that often exceed European and Belgian legal threshold values for Cd (Directive 2002/32/EC of the European Parliament and of the Council of 7 May 2002; Commission Regulation no. 1881/2006; Meers *et al*., 2010; Ruttens *et al*., 2011).

In this metal-contaminated Campine region, a large complex of field plots (∼ 10 ha) situated in Lommel (Belgium) is reserved for phytoremediation research conducted by Hasselt University, Ghent University and the Research Institute for Nature and Forest (INBO) (Meers *et al*., 2010). The soil has a ‘sand’ texture according to the USDA triangle (Meers *et al*., 2010). Mean pseudo-total soil concentrations of Cd and Zn (mg kg^−1^ soil) for the entire test field are respectively 6.5 ± 0.8 and 377 ± 69 (Van Slycken *et al*., 2013). Due to liming, overall pH-H_2_O (6.6 ± 0.2) became suited for growing short rotation forestry (Van Slycken *et al*., 2013). This ongoing phytoremediation research concentrates on screening different plant species, clones and cultivars for their biomass productivity, metal extraction capacity and their subsequent biomass valorization possibilities. In this framework, eight different commercially available willow clones and a number of experimental clones were planted and investigated for their phytoextraction potential (Van Slycken *et al*., 2013). It turned out that the appropriate plant selection is a first crucial step for a successful application of phytoextraction since the biomass production as well as the metal accumulation capacity of the tested clones differed significantly (Van Slycken *et al*., 2013). When for example the willow clones ‘Belgisch Rood (BR)’ (*Salix* × *rubens* var. *basfordiana*) and ‘Tora (TO)’ (*Salix schwerinii* × *Salix viminalis*) are compared (Table 1), the TO willow clones show a Cd and Zn removal capacity (g ha^−1^) that is twice as high as that for BR clones. Both, the higher biomass production (ton ha^−1^) and metal uptake capacity (mg kg^−1^ DW) make the TO willow clone the favourable clone to select for phytoextraction applications.

**Table 1:** Cd and Zn accumulation capacity of Belgisch Rood (BR) and Tora (TO) short rotation willow cuttings after 2 years of growth and after 3 years of growth

	BR	TO
Harvested shoot biomass (ton ha^−1^) after 2 years	1.17 ± 0.37	1.45 ± 0.20
Harvested shoot biomass (ton ha^−1^) after 3 years	5.40 ± 0.29	9.00 ± 1.40
Total_(aqua regia)_ Cd in soil [mg (kg DW)^−1^]	6.60 ± 1.10	6.50 ± 0.61
Cd in the shoot [mg (kg DW)^−1^] after 2 years	19.42 ± 1.65	26.97 ± 6.79
Cd in the shoot [mg (kg DW)^−1^] after 3 years	23.74 ± 1.72	30.50 ± 3.90
Cd removal (g ha^−1^) after 2 years	22.72 ± 0.48	39.11 ± 1.36
Cd removal (g ha^−1^) after 3 years	128.20 ± 0.50	274 ± 0.55
Total_(aqua regia)_ Zn in soil [mg (kg DW)^−1^]	376.00 ± 72.01	359.02 ± 32.00
Zn in the shoot [mg (kg DW)^−1^] after 2 years	679.20 ± 96.53	1046.03 ± 238.83
Zn in the shoot [mg (kg DW)^−1^] after 3 years	918.43 ± 106.70	1268.59 ± 82.43
Zn removal (g ha^−1^) after 2 years	794.66 ± 35.72	1516.74 ± 47.77
Zn removal (g ha^−1^) after 3 years	4959.52 ± 30.94	11417.31 ± 115.40

Data shown in this table are calculated based on the data from Van Slycken and colleagues (2013).

Beside the importance of the selection of the appropriate plant, it is also known that plant-associated bacteria may play an important role during metal phytoextraction (Weyens *et al*., 2009c; 2010a,b; Sherian *et al*., 2012).

Plant-associated bacteria include endophytic, rhizospheric and phyllospheric bacteria. Endophytic bacteria are defined as those bacteria that colonize the internal tissue of the plant without causing visible external sign of infection or negative effect of their host (Schulz and Boyle, 2006). Since these endophytic bacteria can proliferate inside the plant tissue, they are likely to interact closely with their host, face less competition for nutrients, and are more protected from adverse changes in the environment than bacteria in the rhizosphere or in the phyllosphere (Reinhold-Hurek and Hurek, 1998; Beattie, 2007). The phyllosphere can be referred to as the external regions of the above-ground plant parts, including leaves, stems, blossoms and fruits. Rhizospheric bacteria are living in the direct vicinity of the roots. Therefore, root exudates are believed to have a major influence on the diversity of microorganisms within the rhizosphere (Lemanceau *et al*., 1995).

Rhizosphere bacteria, phyllosphere bacteria as well as endophytes can contribute to an improved phytoremediation efficiency (Valls and de Lorenzo, 2002; Lebeau *et al*., 2008; Kidd *et al*., 2009; Mastretta *et al*., 2009; Rajkumar *et al*., 2009; Taghavi *et al*., 2009; van der Lelie *et al*., 2009; Weyens *et al*., 2009b,c,d; 2010c; 2011; Becerra-Castro *et al*., 2011; 2012).

At first, plant-associated bacteria can have beneficial effects on plant growth and development and in this way reduce metal phytotoxicity during metal phytoextraction. Moreover, a more extended root system can also contribute to an increased metal ‘foraging’ and by consequence uptake. Mechanisms of plant growth promotion by plant-associated bacteria vary greatly, and have been broadly categorized into two groups, direct and indirect (Kloepper *et al*., 1989).

Direct plant growth promoting mechanisms involve (i) nitrogen fixation by free living endophytic bacteria, especially diazotrophs (Brusamarello-Santos *et al*., 2012), (ii) the supply of unavailable nutrients such as phosphorus and other mineral nutrients, (iii) production of regulators of plant growth and development such as auxins, cytokinins and gibberelines, and (iv) suppression of ethylene production by 1-aminocyclopropane-1-carboxylate (ACC) deaminase activity (Glick *et al*., 2007). Plant-associated bacteria can indirectly benefit plant growth by inhibiting growth or activity of plant pathogens. This inhibition can be attributed to any of a variety of mechanisms including the competition for space and nutrients, the production of biocontrol agents (e.g. antibiotics, antifungal metabolites), the induction of systemic resistance and the depletion of iron from the rhizosphere through the production of siderophores (Glick, 2010).

Beside their plant growth promoting effects, plant-associated bacteria can also be exploited to enhance the plant availability of metals. Rhizosphere bacteria and root endophytes that produce organic acids, siderophores or other metal-chelating agents can increase metal availability and uptake for themselves, but also for their host plant (Saravanan *et al*., 2007; Braud *et al*., 2009). Furthermore, endophytes equipped with a bacterial Cd resistance involving a three-component efflux system such as czc or czr (Hassan *et al*., 1999) can trigger Cd sequestration, making it less plant available and by consequence reducing Cd phytotoxicity. On top of that, bacteria with the above-described characteristics are frequently naturally abundant in plants growing on metal-contaminated sites (Sessitsch and Puschenreiter, 2008).

Taking into consideration (i) that the metal accumulation capacity of various clones of plants of the same species can be significantly different (Table 1; Van Slycken *et al*., 2013) and (ii) that plant-associated bacteria play an important role during metal phytoextraction (Weyens *et al*., 2009b,c), the question arises if the differences in metal accumulation capacity between these clones might, at least partly, be related to differences in their associated bacterial population.

To investigate this research question, all cultivable bacteria were isolated from the TO and BR willow clones, which possess clearly different metal accumulation capacities (Table 1) and that are growing on the above-mentioned metal-contaminated experimental site in Lommel (Belgium). All isolated bacteria were characterized genotypically by 16SrDNA identification and diversity analysis, and phenotypically by testing some of their plant growth promoting characteristics and their metal (Cd and Zn) resistance.

## Results and discussion

Bacteria were isolated from BR and TO willow clones growing on the metal-contaminated site in Lommel and a comparison of their genotypic and phenotypic characteristics was made. Since the phenotypic characterization tests are only applicable for cultivable bacteria, the bacterial isolation and characterization performed in this work concentrated on the cultivable strains.

### Isolation of bacteria associated with Belgisch Rood and Tora

All cultivable bacteria were isolated from the rhizosphere, the roots and the shoots of BR and TO clones. For the BR clone, the number of cultivable bacteria recovered was the highest in the rhizosphere [6.02 × 10^6^ colony-forming units (cfu) per gram fresh weight], slightly lower in the roots (1.85 × 10^6^ cfu per gram fresh weight) and again lower in the shoots (2.95 × 10^5^ cfu per gram fresh weight). In general, this decrease in bacterial number from the rhizosphere to the shoot is considered as an indication that colonization was mainly taking place through the roots (Weyens *et al*., 2009a; 2011). In case of the TO clones, the number of isolated bacteria was also decreasing from the rhizosphere (3.62 × 10^6^ cfu per gram fresh weight) to the roots (1.74 × 10^5^ cfu per gram fresh weight), suggesting that, also part of the endophytic bacteria entered the plant through the roots. Further, a remarkably high number (even higher than in the roots) of cultivable strains was isolated from the shoots of TO (6.65 × 10^6^ cfu per gram fresh weight), indicating that colonization through the aerial parts might also be a possibility and/or that obligate endophytes residing in the cuttings could represent a significant part of the bacterial population.

### Genotypic characterization

**Figure 1:**
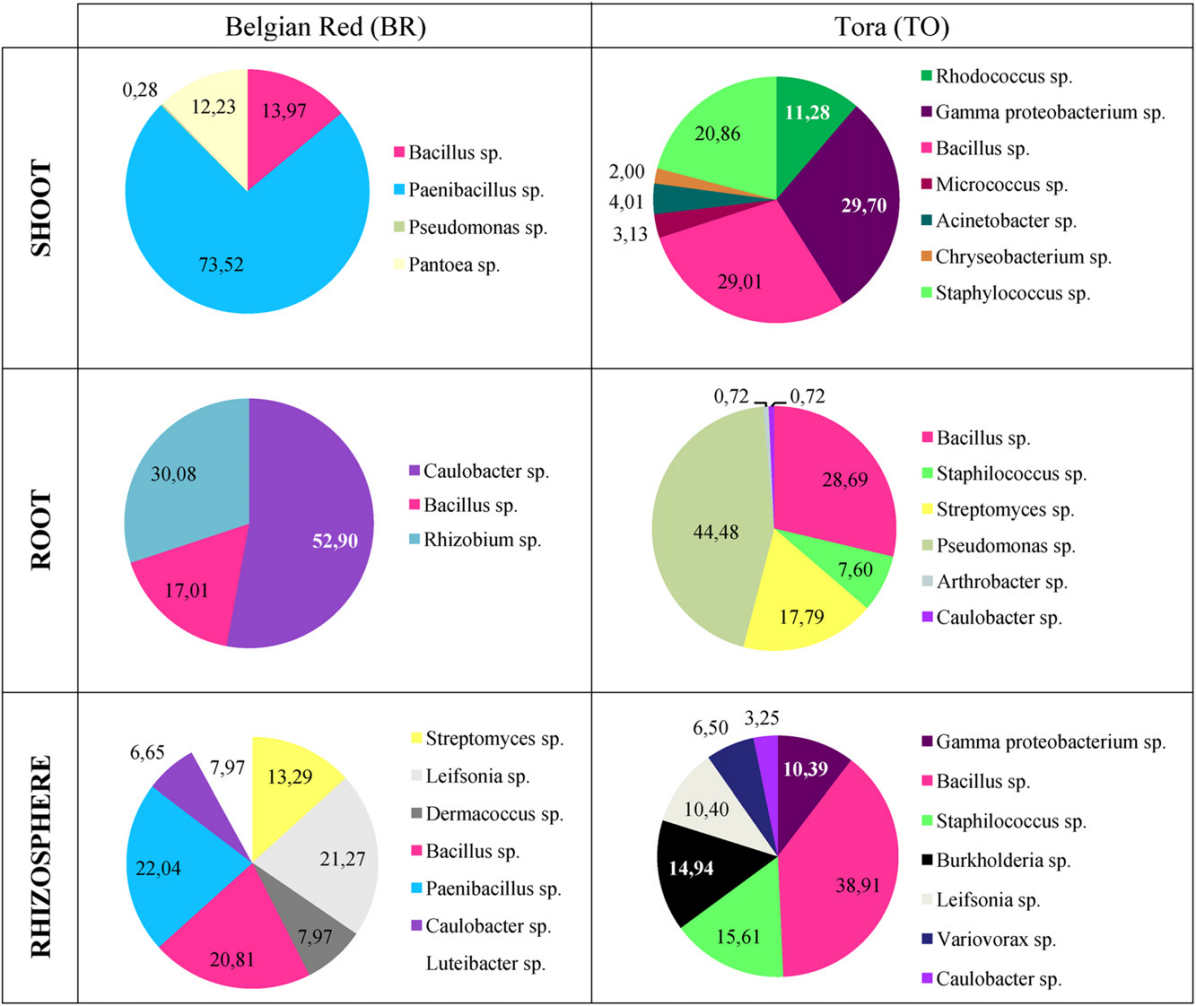
Diversity of cultivable bacteria in the rhizosphere, the roots and the shoot of willow clones of BR (left side) and of TO (right side) growing on a metal-contaminated soil. Numbers indicate the relative abundance, expressed as a percentage of the total number of isolates that are present in the rhizosphere, the roots and the shoot.

After purification, all morphologically different bacteria were identified by means of 16SrDNA ARDRA analysis followed by 16SrDNA sequencing and blasting (Weyens *et al*., 2009a). To visualize the different bacterial species that are associated with BR and TO clones, a distinction was made between rhizosphere bacteria, root endophytes and shoot endophytes (Fig. 1). The relative abundance of each different species was expressed as a percentage of the total number of isolates per gram fresh weight of soil in the rhizosphere, and tissue of roots or shoots of BR and TO clones.

In the rhizosphere associated with BR willow clone, *Paenibacillus* sp., *Leifsonia* sp. and *Bacillus* sp. were the most abundant accounting for respectively 22.04%, 21.27% and 20.81% of the isolated strains. The remaining part of the isolated BR-associated rhizosphere bacteria consisted of *Streptomyces* sp. (13.29%), *Dermacoccus* sp. (7.97%), *Luteibacter* sp. (7.97%) and *Caulobacter* sp. (6.65%). The cultivable bacterial population present in the rhizosphere of TO was dominated by *Bacillus* sp. (38.91%). Next to *Bacillus* sp., *Staphilococcus* sp. (15.61%) and *Burkholderia* sp. (14.94%) also made up a significant part. The remaining 30% of the collection consisted of *Leifsonia* sp. (10.40%), *Variovorax* sp. (6.50%), *Caulobacter* sp. (3.25%) and the remaining 10.39% could only be identified up to the level of *Gammaproteobacteria*.

The root endophytes isolated from BR were dominated by *Caulobacter* sp. (52.90%). Beside these *Caulobacter* sp., only *Rhizobium* sp. (30.08%) and Bacillus sp. (17.01%) could be recovered from the roots of BR. In the roots of TO, *Pseudomonas* sp. (44.48%) and *Bacillus* sp. (28.69%) formed the majority of the isolated endophytes. The residual 27% of the TO-associated root endophytes was consisted of *Streptomyces* sp. (17.79%), *Staphylococcus* sp. (7.60%) and *Arthrobacter* sp. and *Caulobacter* sp. accounted each for a minority of 0.72%.

In the BR shoot-associated cultivable population, *Paenibacillus* sp. (73.52%) made up the majority of the total number of isolates. The remaining minority of the BR-associated shoot endophytes consisted of *Bacillus* sp. (13.97%), *Pantoea* sp. (12.23%) and *Pseudomonas* sp. (0.28%). The shoot endophytes recovered from TO were mainly represented by gamma Protebacteria (29.70%) (identification reliable up to this level), *Bacillus* sp. (29.01%), *Staphylococcus* sp. (20.86%) and *Rhodococcus* sp. (11.28%). *Acinetobacter* sp. (4.01%), *Micrococcus* sp. (3.13%) and *Chryseobacterium* sp. (2.00%) only accounted for a small minority.

Taking a closer look to these results, a distinction can be made between willow-associated bacteria that comprise a high clone or compartment specificity, and others that show no specificity at all. For example, the isolated *Bacillus* sp. can be classified as non-specific, since they colonized both willow clones and were recovered from all plant compartments. Other species showed to be only clone-specific: *Paenibacillus* sp. only colonized BR, but could be isolated from rhizosphere and shoot samples while *Staphylococcus* sp. was isolated from rhizosphere, root and shoot samples, but exclusively associated with TO. On the contrary, *Leifsonia* sp. were associated with both clones, but could not enter inside the willow plants and by consequence can be considered as strict rhizosphere strains.

**Figure 2:**
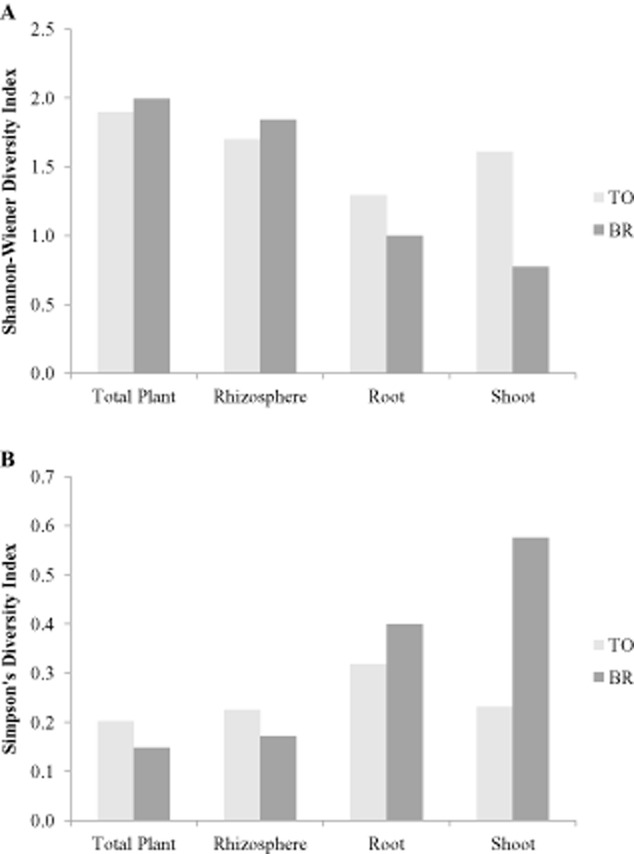
Shannon–Wiener Diversity Index (A) and Simpson's Diversity Index of the cultivable bacteria associated with the total plant (rhizosphere + root + shoot-associated bacteria), the rhizosphere, the root and the shoot of TO and BR willow clones growing on a metal-contaminated site. Shannon–Wiener Diversity Index: the higher the index, the higher the diversity. Simpson's Diversity Index: the lower the index, the higher the diversity.

In order to compare the bacterial diversity that is associated with both clones, the Shannon–Wiener and the Simpson's diversity indices were calculated (Fig. 2). These indices can be easily interpreted: the higher the Shannon–Wiener index, the higher the diversity while a higher Simpon's index, corresponds with a lower diversity. When the total cultivable bacterial population is considered, the diversity was only slightly higher for the BR clone. The same is true for all isolated rhizosphere bacteria. However, in the roots and certainly in the shoots, the difference in bacterial diversity between BR and TO clones becomes more obvious with the highest diversity in the TO clones. Wittebolle *et al*. (2009) demonstrated that the functioning of a bacterial community with a higher diversity is more resistant to environmental stress than a bacterial community with a lower diversity. Knowing this, we might hypothesize that the higher endophytic bacterial diversity observed in the TO clone might result in a better functioning of the endophytic TO-associated bacterial community under the present metal stress which might contribute to a better growth and development of their host plant.

### Phenotypic characterization

All strains isolated from Tora and Belgisch Rood were screened for their potential to assist their host plant during metal phytoextraction by testing their plant growth promoting capacity and their metal resistance.

Plant-associated bacteria can promote the growth and development of their host by means of many different mechanisms. For this work, three generally applied standard plant growth promotion tests were selected, namely: the production of siderophores, organic acids and indole-3-acetic acid (IAA).

Siderophores are complex molecules that bind Fe^3+^ and render it available for reduction into the Fe^2+^, which is preferred by bacteria and plants. The formed bacterial Fe^3+^–siderophore complexes will facilitate uptake of iron into bacteria, but there is also evidence that several plant species can recognize and take up bacterial Fe^3+^–siderophore complexes (Sharma and Johri, 2003; Katiyar and Goel, 2004). Bacteria that produce organic acids may also contribute to an improved uptake of essential mineral nutrients that are frequently limiting in soil. The (plant)unavailable inorganic phosphate can, e.g. be solubilized by bacteria through the release of organic acids, such as gluconic acid and 2-ketogluconic acid. Beside improving the availability of essential mineral nutrients, the production of siderophores and organic acids also results in an improved availability of toxic metals by means of the same mechanisms. The auxin IAA is the most studied phytohormone produced by plant-associated bacteria. It is known to contribute to plant growth and development (Vessey, 2003) by increasing root growth and root length, and has also been associated with proliferation and elongation of root hairs (Taghavi *et al*., 2009). The extended root system that can be induced by bacterial IAA production (Weyens *et al*., 2010c) will in the first place result in an improved biomass production, but beside of that it will enable the plant to ‘forage’ a bigger soil volume and by consequence more essential and non-essential elements.

To compare the potential plant growth promoting capacity of the BR- and the TO-associated bacteria, the total percentages of bacteria capable to produce siderophores, organic acids or IAA were calculated for each compartment (Table 2).

**Table 2:** Potential plant growth promoting characteristics of cultivable strains isolated from the shoot, the roots and the rhizosphere of BR and TO willow clones growing on metal-contaminated soil

	BR					TO				
Identification	cfu/g	SID	OA	IAA	Identification	cfu/g	SID	OA	IAA
Shoot	*Bacillus pumilus*	1.14E+04	+	−	+	Unc. rhodococcus	7.50E+05	−	−	−
*Bacillus pumilus*	2.29E+04	−	−	+	Unc. γ-proteobacterium	1.98E+06	−	−	+
*Bacillus pumilus*	3.81E+03	−	−	+	*Bacillus cereus*	7.50E+05	−	−	+
*Bacillus pumilus*	1.24E+03	−	+	−	*Bacillus cereus*	2.08E+05	+	+	+
*Bacillus pumilus*	1.24E+03	−	+	−	*Bacillus cereus*	1.38E+05	−	−	−
*Bacillus pumilus*	6.20E+02	−	−	−	*Bacillus cereus*	7.75E+01	+	−	+
*Paenibacillus lactis*	2.15E+05	−	+	−	*Bacillus subtilis*	7.75E+01	+	−	+
*Paenibacillus lactis*	8.39E+02	−	−	+	*Bacillus pumilis*	7.75E+01	−	+	+
*Paenibacillus lactis*	8.39E+02	−	−	−	*Bacillus* sp.	7.50E+05	−	−	−
*Pseudomonas syringae*	8.12E+02	−	−	+	*Bacillus* sp.	6.25E+04	−	+	−
*Pantoea agglomerans*	2.89E+04	+	−	−	*Bacillus* sp.	2.08E+04	+	−	−
*Pantoea agglomerans*	7.22E+03	+	+	−	*Bacillus* sp.	7.75E+01	+	+	−
					*Micrococcus* sp.	2.08E+05	−	−	+
					*Acinetobacter johnsonii*	2.67E+05	−	−	+
					*Chryseobacterium hominis*	1.33E+05	−	−	−
					*Staphylococcus pasteuri*	1.39E+06	−	+	−
Total (%)	2.95E+05	16.11	76.24	13.48	Total (%)	6.65E+06	3.45	24.94	51.25
Root	*Caulobacter* sp.	7.85E+05	−	−	−	*Bacillus cereus*	2.13E+04	−	+	+
*Caulobacter* sp.	1.96E+05	+	−	−	*Bacillus cereus*	1.50E+04	+	−	−
*Bacillus pumilus*	2.10E+05	−	+	−	*Bacillus* sp.	1.10E+04	+	+	−
*Bacillus pumilus*	5.26E+04	+	−	−	*Bacillus* sp.	2.75E+03	−	+	−
*Bacillus pumilus*	5.26E+04	−	−	−	*Staphylococcus* sp.	1.25E+03	+	−	−
*Rhizobium radiobacter*	5.58E+05	−	−	−	*Staphylococcus* sp.	3.75E+03	−	−	−
					*Staphylococcus* sp.	5.75E+03	−	+	+
					*Staphylococcus* sp.	1.25E+03	+	−	+
					*Staphylococcus* sp.	1.25E+03	−	+	−
					*Streptomyces* sp.	1.73E+04	−	+	−
					*Streptomyces punicolor*	1.38E+04	+	+	−
					*Pseudomonas* sp.	5.75E+04	+	+	+
					*Pseudomonas* sp.	1.88E+04	+	−	+
					*Pseudomonas* sp.	1.25E+03	+	−	−
					*Arthrobacter gandavensis*	1.25E+03	−	−	+
					*Caulobacter* sp.	1.25E+03	−	−	+
Total (%)	1.85E+06	13.42	11.34	0.00	Total (%)	1.74E+05	68.72	74.89	61.41
Rhizosphere	*Streptomyces* sp.	8.00E+05	−	−	−	Unc. γ proteobacteria	2.59E+05	+	−	−
*Leifsonia* sp.	1.28E+06	−	−	−	Unc. γ proteobacteria	1.18E+05	+	+	−
*Dermacoccus* sp.	4.80E+05	−	−	−	*Bacillus* sp.	2.35E+05	+	+	+
*Bacillus pumilus*	8.35E+05	−	−	−	*Bacillus* sp.	2.82E+05	+	+	−
*Bacillus pumilus*	4.18E+05	+	−	−	*Bacillus* sp.	4.24E+05	−	−	+
*Paenibacillus lactis*	1.33E+06	−	−	−	*Bacillus* sp.	1.18E+05	−	−	−
*Caulobacter* sp.	2.00E+05	+	−	−	*Bacillus* sp.	3.53E+05	+	−	−
*Caulobacter* sp.	2.00E+05	−	−	+	*Staphylococcus epidermis*	1.41E+05	+	−	+
*Luteibacter rhizovicinus*	4.80E+05	−	−	+	*Staphylococcus capitis*	3.06E+05	+	+	−
					*Staphylococcus* sp.	1.18E+05	−	+	−
					*Burkholderia pyrrocinia*	1.41E+05	+	−	−
					*Burkholderia* sp.	4.00E+05	+	−	−
					*Leifsonia* spec.	3.76E+05	+	−	+
					*Variovorax* sp.	2.35E+05	+	+	−
					*Caulobacter* sp.	1.18E+05	−	−	−
Total (%)	6.02E+06	10.26	0.00	11.30	Total (%)	3.62E+06	78.56	35.73	32.46

cfu/g: colony-forming units per gram fresh weight; SID: siderophore production; OA: organic acid production; IAA: indole-3-acetic acid production. −: negative test; +: positive test. Total: cfu/g: total amount of isolated bacteria in shoot, root or rhizosphere; SID, OA, IAA, Cd (0.4), Cd (0.8), Zn (0.6) and Zn (1.0): percentage of bacteria associated with the shoot, root and rhizosphere that tested positive.

In the rhizosphere of the BR willow clone, 10.26% of the strains showed capable to produce siderophores, none of the isolated strains produced organic acids and 11.30% tested positive for IAA production. When the TO-associated rhizosphere bacteria are considered, the amount of potentially plant growth promoting strains is remarkably higher than for the BR-associated rhizosphere strains: 78.56%, 35.73% and 32.46% produced respectively siderophores, organic acids and IAA. The same difference was observed for the isolated root endophytes: only 13.42%, 11.34% and even 0% of BR-associated strains could produce respectively siderophores, organic acids and IAA, while 68.72% of the root endophytes recovered from TO produced siderophores, 74.89% organic acids and 61.41% IAA. However, when the potential growth promoting characteristics of both shoot-associated bacteria are compared, only the percentage of IAA producing bacteria is higher for the TO clones (51.25%) than for the BR clones (13.48%) while the opposite is observed for the organic acid production and IAA production tests where respectively 76.24% and 13.48% tested positive for the BR clone and only 24.94% and 51.25% for the TO clone. Nevertheless, the total amount of siderophore and organic acid producing bacteria is higher in the shoots of TO (respectively 3.45% of 6.65 × 10^6^ = 22.94 × 10^4^ and 24.94% of 6.65 × 10^6^ = 16.59 × 10^5^) than in the shoots of BR (respectively 16.11% of 2.95 × 10^5^ = 47.52 × 10^3^ and 76.24% of 2.95 × 10^5^ = 22.49 × 10^4^).

It is also remarkable that the TO-associated bacteria with the highest potential to promote plant growth (a positive test for siderophores, organic acids and IAA) are always belonging to the genus that is dominating the population (Table 2 and Fig. 1). In the rhizosphere, *Bacillus* sp. were the most dominant of the bacterial population and it was a *Bacillus* strain that tested positive for all growth promoting tests; in the roots, the *Pseudomonas* sp. represented almost half of the cultivable bacterial population and a *Pseudomonas* strain scored best for the growth promoting tests; in the shoots, a *Bacillus* strain had the highest plant growth promoting potential and also here *Bacillus* sp. were one of the most dominant genera.

Beside their plant growth promoting capacity, the metal resistance of the isolated strains is also an important feature to further reduce metal toxicity for their host plant. Plant-associated bacteria that are equipped with a metal sequestration system are capable of ‘precipitating’ metals on their cell walls reducing the amount of bioavailable toxic metals inside the plant. This results in a decreased metal toxicity for themselves as well as for their host plant and it even can contribute to an increased metal translocation efficiency (Lodewyckx *et al*., 2001; Mastretta *et al*., 2009).

When the amount of isolated Cd- and Zn-resistant bacteria is compared between the two willow clones, only a small part of the BR-associated bacteria turned out to be metal-resistant while a very significant fraction of the TO-associated bacteria showed resistance to Cd and Zn (Table 3).

**Table 3:** Metal resistance, possibly resulting in decreased phytotoxicity, of cultivable strains isolated from the shoot, the roots and the rhizosphere of BR and TO willow clones growing on metal-contaminated soil

	BR						TO					
Identification	cfu/g	Cd (0.4)	Cd (0.8)	Zn (0.6)	Zn (1.0)	Identification	cfu/g	Cd (0.4)	Cd (0.8)	Zn (0.6)	Zn (1.0)
Shoot	*Bacillus pumilus*	1.14E+04	−	−	−	−	Unc. rhodococcus	7.50E+05	−	−	−	−
	*Bacillus pumilus*	2.29E+04	−	−	−	−	Unc. γ-proteobacterium	1.98E+06	−	−	−	−
	*Bacillus pumilus*	3.81E+03	−	−	+	−	*Bacillus cereus*	7.50E+05	+	−	−	−
	*Bacillus pumilus*	1.24E+03	−	−	+	+	*Bacillus cereus*	2.08E+05	−	−	−	−
	*Bacillus pumilus*	1.24E+03	−	−	−	−	*Bacillus cereus*	1.38E+05	+	−	+	+
	*Bacillus pumilus*	6.20E+02	−	−	−	−	*Bacillus cereus*	7.75E+01	−	−	+	+
	*Paenibacillus lactis*	2.15E+05	−	−	−	−	*Bacillus subtilis*	7.75E+01	−	−	−	−
	*Paenibacillus lactis*	8.39E+02	−	−	−	−	*Bacillus pumilis*	7.75E+01	+	−	+	+
	*Paenibacillus lactis*	8.39E+02	−	−	−	−	*Bacillus* sp.	7.50E+05	−	−	+	+
	*Pseudomonas syringae*	8.12E+02	−	−	−	−	*Bacillus* sp.	6.25E+04	−	−	−	−
	*Pantoea agglomerans*	2.89E+04	−	−	−	−	*Bacillus* sp.	2.08E+04	−	−	−	−
	*Pantoea agglomerans*	7.22E+03	−	−	−	−	*Bacillus* sp.	7.75E+01	−	−	+	−
							*Micrococcus* sp.	2.08E+05	+	−	−	−
							*Acinetobacter johnsonii*	2.67E+05	−	−	−	−
							*Chryseobacterium hominis*	1.33E+05	−	−	−	−
							*Staphylococcus pasteuri*	1.39E+06	−	−	−	−
Total (%)	2.95E+05	0.00	0.00	1.71	0.42	Total (%)	6.65E+06	16.48	0.00	13.35	13.35
Root	*Caulobacter* sp.	7.85E+05	−	−	−	−	*Bacillus cereus*	2.13E+04	+	−	+	+
	*Caulobacter* sp.	1.96E+05	−	−	−	−	*Bacillus cereus*	1.50E+04	−	−	+	+
	*Bacillus pumilus*	2.10E+05	−	−	−	−	*Bacillus* sp.	1.10E+04	−	−	+	−
	*Bacillus pumilus*	5.26E+04	−	−	−	−	*Bacillus* sp.	2.75E+03	−	−	+	+
	*Bacillus pumilus*	5.26E+04	−	−	−	−	*Staphylococcus* sp.	1.25E+03	+	−	−	−
	*Rhizobium radiobacter*	5.58E+05	−	−	−	−	*Staphylococcus* sp.	3.75E+03	−	−	−	−
							*Staphylococcus* sp.	5.75E+03	−	−	−	−
							*Staphylococcus* sp.	1.25E+03	+	+	+	−
							*Staphylococcus* sp.	1.25E+03	−	−	+	−
							*Streptomyces* sp.	1.73E+04	−	−	+	−
							*Streptomyces punicolor*	1.38E+04	+	+	+	−
							*Pseudomonas* sp.	5.75E+04	+	+	−	−
							*Pseudomonas* sp.	1.88E+04	+	+	−	−
							*Pseudomonas* sp.	1.25E+03	+	−	+	−
							*Arthrobacter gandavensis*	1.25E+03	−	−	−	−
							*Caulobacter* sp.	1.25E+03	−	−	−	−
Total (%)	1.85E+06	0.00	0.00	0.00	0.00	Total (%)	1.74E+05	66.00	52.37	48.64	22.38
Rhizosphere	*Streptomyces* sp.	8.00E+05	−	−	−	−	Unc. γ-proteobacteria	2.59E+05	−	−	+	−
	*Leifsonia* sp.	1.28E+06	+	+	−	−	Unc. γ-proteobacteria	1.18E+05	−	−	+	−
	*Dermacoccus* sp.	4.80E+05	−	−	−	−	*Bacillus* sp.	2.35E+05	−	−	+	−
	*Bacillus pumilus*	8.35E+05	−	−	−	−	*Bacillus* sp.	2.82E+05	−	−	+	+
	*Bacillus pumilus*	4.18E+05	+	+	−	−	*Bacillus* sp.	4.24E+05	−	−	−	−
	*Paenibacillus lactis*	1.33E+06	−	−	−	−	*Bacillus* sp.	1.18E+05	−	−	−	−
	*Caulobacter* sp.	2.00E+05	−	−	−	−	*Bacillus* sp.	3.53E+05	+	−	+	+
	*Caulobacter* sp.	2.00E+05	−	−	−	−	*Staphylococcus epidermis*	1.41E+05	+	+	−	−
	*Luteibacter rhizovicinus*	4.80E+05	−	−	−	−	*Staphylococcus capitis*	3.06E+05	−	−	−	−
							*Staphylococcus* sp.	1.18E+05	−	−	+	−
							*Burkholderia pyrrocinia*	1.41E+05	+	+	+	+
							*Burkholderia* sp.	4.00E+05	+	+	+	−
							*Leifsonia* spec.	3.76E+05	+	+	+	+
							*Variovorax* sp.	2.35E+05	−	−	+	−
							*Caulobacter* sp.	1.18E+05	−	−	−	−
	Total (%)	6.02E+06	28.20	28.20	0.00	0.00	Total (%)	3.62E+06	38.95	29.22	69.49	31.81

cfu/g: colony-forming units per gram fresh weight; 284 medium with addition of carbon mix and respectively 0.4 mM CdSO_4_, 0.8 mM CdSO_4_, 0.6 mM ZnSO_4_ and 1 mM ZnSO_4_.

+: good growth; −: no growth. Total: cfu/g: total amount of isolated bacteria in shoot, root or rhizosphere; Cd (0.4), Cd (0.8), Zn (0.6) and Zn (1.0): percentage of bacteria associated with the shoot, root and rhizosphere that tested positive.

From the rhizosphere of BR, no Zn-resistant bacterial strains were isolated and 28.2% were highly Cd-resistant (low and high [Cd]) while the majority (69.49%) of rhizosphere bacteria associated with TO were able to grow on the low Zn concentrations, 31.81% on the high Zn concentrations, 38.95% on the low and 29.22% on the high Cd concentrations. Not a single strain of the BR-associated root endophytes was Zn- or Cd-resistant while the amount of Zn-resistant TO root endophytes was quite high (low [Zn]: 48.64% and high [Zn]: 22.38%) and the Cd-resistant root endophytes even represented more than half of the population (low [Cd]: 66.00% and high [Cd]: 52.37%).

The metal-resistant strains of the BR-associated shoot endophytes only represented a very small minority: 0% of the population was Cd-resistant, 1.71% resisted the low Zn concentration and 0.42% the high Zn concentration. The amount of metal-resistant TO-associated bacteria is remarkably lower in the shoots than in the rhizosphere and the roots: 13.35% was highly Zn-resistant (low and high [Zn]) and 16.48% could only grow on the low Cd concentration.

In general, the amount of metal-resistant bacteria with the potential to promote plant growth is remarkably higher in the TO willow clones. These metal-resistant, plant growth promoting bacteria can play an important role in improving biomass production, reducing metal phytotoxicity and increasing metal availability for the plant (Weyens *et al*., 2009b,c). Therefore, we hypothesize that the difference in their abundance between the two tested willow clones might be related to the higher biomass production and metal accumulation capacity of the TO clone.

## Conclusion

In this study, the cultivable bacteria associated with the willow clones BR and TO were compared. TO was shown to possess a twice as high metal removal capacity in comparison with BR. The TO endophytic bacterial population comprises a higher bacterial diversity as well as a significantly higher amount of bacteria with the potential to improve metal phytoextraction efficiency. The TO bacterial population have better PGPR abilities, higher metal phytoextraction capacity and higher tolerance to Cd and Zn. By consequence, the obtained data are in agreement with our hypothesis that the higher metal phytoextraction capacity of the TO willow clone might be, at least partly, related to differences in the characteristics of their associated bacterial populations. Although it is known that this ‘cultivable’ bacterial population only represents a small fraction of the total bacterial population, it can be expected that the differences in characteristics that we observed between both cultivable bacterial populations is representative for the total population. In future experiments, this will be further investigated; however, a characterization of the total population would be limited to the genotypic level.

Given that there is a relation between the metal phytoextraction capacity of the willow clones and their associated bacterial population, it would be interesting to know what comes first. Is the presence of a high amount of metal-resistant plant growth promoting bacteria with a high diversity the reason why the TO clones are better metal extractors or is the higher metal uptake in the TO clones causing a shift in their bacterial population? This will be explored in a future experiment in which the metal phytoextraction capacity of the BR clones will be evaluated before and after inoculation of the cultivable TO-associated bacterial population.

In conclusion, providing an innovative insight in what are key factors to optimize metal phytoextraction and in how plant-associated bacteria might play a role, this work will contribute to the break-through of large-scale applications of metal phytoextraction.

## Experimental procedures

### Site background and plant selection

The experimental field site was a former maize field that is located nearby metal smelters in Lommel (Campine region) in Flanders, Belgium (51°12′41″N, 5°14′32″E). Historic activities of these smelters resulted in a diffuse metal [Cd: 6.5 ± 0.8; Zn: 377 ± 70; Pb: 198 ± 17 mg kg^−1^ DW (*aqua regia*)] contamination in this region (Vangronsveld *et al*., 1995; Hogervorst *et al*., 2007). In previous work, eight commercially available willow clones were selected and planted on this site in order to screen their metal phytoextraction potential (Van Slycken *et al*., 2013). For this study, the willow clones ‘Belgisch Rood (BR)’ (*Salix* × *rubens* var. *basfordiana*) and ‘Tora (TO)’ (*Salix schwerinii* × *Salix viminalis*) were selected because of the difference in their metal accumulation capacities (Table 1). The harvestable shoot biomass (including the leaves) of TO after 2 as well as after 3 years was significantly higher than for the BR clones. This, together with the higher Cd and Zn concentrations in the harvested shoots of TO, results in a Cd and Zn removal (g ha^−1^) for TO clones that is more or less twice as high as for BR clones.

### Isolation of cultivable willow-associated bacteria

The ultimate aim of this work was to investigate if the different metal accumulation capacities of the TO and BR willow clones can be related to differences in the characteristics of their associated bacteria. Since phenotypic characterization can only be performed for cultivable bacteria, the isolation of the willow-associated bacteria in this work was restricted to the cultivable bacteria.

To isolate the cultivable willow-associated rhizosphere bacteria, roots with the surrounding soil were vortexed, roots were removed, serial dilutions up to 10^−5^ were prepared in 10 mM MgSO_4_ solution and plated on 1/10 diluted 869 solid medium (Mergeay *et al*., 1985), and plates were incubated for 7 days at 30°C.

The cultivable root and shoot endophytes were isolated by applying (i) a chloride surface sterilization, (ii) three rinse steps, (iii) a surface sterility check and (iv) a maceration as described in Weyens and colleagues (2013). Serial dilutions up to 10^−5^ of the obtained bacterial suspension were plated on 1/10 diluted 869 solid medium (Mergeay *et al*., 1985) and plates were incubated for 7 days at 30°C. After incubation, cfu were counted and calculated per gram rhizosphere soil or fresh root or shoot weight. All morphologically different bacteria were purified three times on 1/10 diluted 869 solid medium (Mergeay *et al*., 1985).

To take into account the biological variation and at the same time keeping the experiment practically feasible, for each condition (clone; plant compartment), bacteria were isolated from 10 biologically independent samples that were pooled together in one mixed sample.

### Genotypic characterization of isolated bacteria

After total genomic DNA from all morphologically different purified bacteria was extracted using the DNeasy® Blood and Tissue kit (Qiagen), the 16SrDNA was amplified by a PCR with the universal 1392R (5′-ACGGGCGGTGTGTGTRC-3′) and the Bacteria-specific 26F (5′-AGAGTTTGATCCTGGCTCAG-3′) primers as described previously by Weyens and colleagues (2009a). To distinguish the different 16SrDNA samples, PCR products of the 16SrDNA amplification were directly used for the Amplified rDNA Restriction Analysis (ARDRA) that was performed with the *Hpy*CH4 IV restriction enzyme (Weyens *et al*.*,* 2009a). The 16SrDNA PCR products of one representative strain per group were purified according to the QIAquick 96 PCR Purification Kit (Qiagen, Valencia, CA, USA) and quantified spectrophotometrically using a Nanodrop spectrophotometer (ND-1000, Isogen Life Science). These purified 16S rRNA genes were sent for sequencing by Macrogen (Korea) under BigDye^TM^ terminator cycling conditions with an Automatic Sequencer 3730XL. After constructing the consensus sequences with Staden Package, sequence match at the Ribosome Database Project II (http://rdp.cme.msu.edu/seqmatch/seqmatch_intro.jsp) was applied for nearest neighbour and species identification.

To compare the (cultivable) bacterial diversity of the TO and BR willow clones, the Shannon–Wiener and the Simpson's diversity indices were calculated for the total isolated plant-associated community and for the different compartments (rhizosphere, root and shoot). Calculations of both indices take into account the species richness as well as their relative abundance.

### Phenotypic characterization of isolated bacteria

All purified morphologically different bacteria were screened for (i) some plant growth promoting features, more exactly their capability to produce siderophores (Sid), organic acids (OA) and the auxin IAA and (ii) for their Cd and Zn resistance.

The applied screening assays to evaluate the plant growth promoting capacity of the isolated strains were all qualitative colorimetric tests that are described in detail in Weyens and colleagues (2013). The siderophore production capacity of the isolated bacterial strains was detected by the universal method of Schwyn and Neilands (1987) using the blue chromium-azurol S (CAS) reagent. The production of organic acids was evaluated according to the colorimetric method of Cunningham and Kuiack (1992) by adding the alzarine red S pH indicator. Bacteria were tested for their IAA production capacity by using the Salkowski assay (adapted from Patten and Glick, 2002).

Finally, bacteria were tested for their Cd and Zn resistance by plating them on selective 284 medium (Weyens *et al*., 2009a) with addition of a carbon mix (CMIX) (per litre of medium: 0.52 g of glucose, 0.35 g of lactate, 0.66 g of gluconate, 0.54 g of fructose and 0.81 g of succinate) and 0, 0.4 and 0.8 mM CdSO_4_ or 0, 0.6 and 1 mM ZnSO_4._ Growth was evaluated after an incubation period of 7 days.

## Conflict of interest

None declared.
